# SQSTM1/p62 regulates HTLV-1 tax mediated NF-κB activation

**DOI:** 10.1186/1742-4690-12-S1-O38

**Published:** 2015-08-28

**Authors:** Aurélien Schwob, Jean-Louis Palgen, Renaud Mahieux, Chloé Journo

**Affiliations:** 1 Équipe Oncogenèse Rétrovirale, Lyon, France; 2 Équipe Labellisée (Ligue Nationale Contre le Cancer), Lyon, France; 3 Centre International de Recherche en Infectiologie, INSERM U1111-CNRS UMR5308, Lyon, France; 4 École Normale Supérieure de Lyon, Lyon, France; 5 Université Lyon 1, LabEx ECOFECT -Eco-evolutionary dynamics of infectious diseases, Lyon, France

## 

HTLV-1-mediated cellular transformation relies on Tax-dependent activation of the NF-κB pathway, which has previously been shown to require Tax poly-ubiquitination and interaction with cellular factors, such as Optineurin (OPTN). The recent identification of OPTN as a selective autophagy receptor sharing high sequence similarities with sequestosome-1 (SQSTM-1/p62), a well-described selective autophagy receptor and NF-κB signaling adaptor, led us to hypothesize that Tax could hijack selective autophagy receptors for an efficient NF-κB activation. Using immunoprecipitation and confocal imaging of endogenous SQSTM/p62 in Tax-expressing cells or in HTLV-1 chronically infected T-cell lines, we showed that Tax interacts with SQSTM-1/p62. This interaction was independent of Tax ubiquitination and of the presence of the Tax PDZ-binding motif. Tax-mediated activation of NF-κB in p62-deficient cells was significantly reduced compared to wild type cells, indicating that SQSTM/p62 is necessary for Tax activity. Surprisingly however, over-expression of SQSTM-1/p62 or induction of autophagy led to a dramatic decrease in the amount of soluble Tax, along with a reduced induction of NF-κB. This suggests that Tax could be a substrate of selective autophagy. Altogether, our results reveal the double-edged consequences of Tax / SQSTM/p62 interaction, with the potentiation of Tax activity and the induction of Tax sequestration. They highlight the complex relationships that this viral protein has.

